# Prediction of Maximum Amplitude With Citrated Rapid Thromboelastography Reaction Time in Cardiac Surgery: A Retrospective Study

**DOI:** 10.7759/cureus.93694

**Published:** 2025-10-02

**Authors:** Takahiro Tamura, Tatsuro Yokoyama

**Affiliations:** 1 Department of Anesthesiology, Nagoya University Graduate School of Medicine, Nagoya, JPN

**Keywords:** citrated rapid thromboelastography, maximum amplitude, observational study, prediction, thromboelastography

## Abstract

Background: Rapid and accurate assessment of coagulation status is essential in cardiac surgery to guide transfusion therapy. The reaction time in citrated rapid thromboelastography (CRT-R) is obtainable within minutes; however, its predictive relationship with maximum amplitude (MA) parameters, particularly platelet- and fibrinogen-related clot strength, remains unclear.

Methods: We retrospectively analyzed TEG6s assays performed in the operating rooms and intensive care units of a single tertiary center from January 2018 to July 2024 in adult patients who underwent cardiac surgery. Pearson coefficients quantified the associations between CRT-R and MA values from the CRT, citrated kaolin with heparinase (CKH), and citrated functional fibrinogen (CFF) assays. Relationships with MA were modeled using an exponential‐decay fit, and CRT-R versus TEG-activated clotting time (TEG-ACT) was modeled linearly. Bootstrap resampling (1,000 iterations) estimated CRT-R thresholds corresponding to clinically relevant MA targets (CRT-MA=48 mm; CFF-MA=12 mm).

Results: Of the 2,453 initially identified paired measurements, 104 were excluded (missing values, n=80; CRT-R >5.5 min, n=24), leaving 2,349 paired measurements for analysis. CRT-R showed inverse associations with MA parameters (absolute Pearson r: CFF-MA 0.6224, CRT-MA 0.6215, CKH-MA 0.6022; all p<0.0001) and a weak association with CKH-R (r=0.2897). CRT-R and TEG-ACT were nearly perfectly linear (r=1.000). Bootstrap-derived CRT-R cutoffs were 1.1858 min (95% CI 1.1597-1.2135) for predicting CRT-MA=48 mm and 1.2832 min (95% CI 1.2487-1.3238) for predicting CFF-MA=12 mm. At these thresholds, CRT-R predicted CRT-MA <48 mm with 49.2% sensitivity and 93.8% specificity and predicted CFF-MA <12 mm with 56.9% sensitivity and 97.0% specificity.

Conclusion: CRT-R, obtainable within minutes of assay initiation, moderately predicts final clot strength, with the strongest link to fibrinogen-dependent CFF-MA. These CRT-R-based thresholds may enable earlier preparation of platelet and fibrinogen therapy within viscoelastic-guided transfusion workflows.

## Introduction

Multiple factors contribute to perioperative coagulation disorders during cardiac surgery, including reduced coagulation factor levels, platelet irregularities, and hyperfibrinolysis. The guidelines of the European Society of Anesthesiology and American Society of Cardiovascular Anesthesiologists strongly recommend the use of viscoelastic tests [1,2] because generic coagulation laboratory tests are not useful in diagnosing and treating perioperative coagulopathy and because differentiating complex conditions using activated partial thromboplastin and prothrombin time, which assess thrombin production using plasma samples, is difficult [3].

Thromboelastography (TEG) is performed at the bedside to assess global coagulability using whole blood, assessing waveforms and reaction time (R) values (which reflect the time to thrombin formation) and maximum amplitude (MA, which reflects the clot strength that is affected by platelets and fibrinogen). R is the time from the start of measurement to initial fibrin formation. It reflects the rate of thrombin production and corresponds to activated partial thromboplastin time and prothrombin time in general coagulation tests. MA is the maximum amplitude of the measured sample, where higher values indicate stronger blood clots. Citrated rapid TEG (CRT) contains both endogenous and exogenous activators and can provide a common measurement in a short time. Citrated kaolin with heparinase (CKH) tests involve heparinase, which antagonizes the action of heparin, and kaolin, an endogenous activator, and can evaluate thrombin-induced coagulation without the action of heparin. Citrated functional fibrinogen (CFF) tests involve GPIIb/IIIa, which inhibits platelet function, allowing clot strength based on fibrinogen alone to be measured. R and MA measurements are conducted concurrently and automatically for CRT, CKH, and CFF and TEG-activated clotting time (ACT). TEG-ACT is converted to the ACT value by subtracting CKH-R from CK-R.

To enable faster preparation and administration of transfusion products, we previously reported that taking steps toward the use of transfusion products, such as the transfusion order and start of dissolution, can be accelerated by predicting the MA using the A10 value obtained with TEG6s, which reflects clot strength 10 minutes after R [4]. However, although the MA evaluation is performed earlier when based on A10, the A10 occurs later when the R value is determined later.

This study aimed to verify whether the CRT-MA, CKH-R, CKH-MA, CFF-MA, and TEG-ACT values could be predicted from the CRT-R value, which is the earliest value determinable with TEG6s, and to evaluate the relationships among any predictable items.

## Materials and methods

Ethics approval

This retrospective study followed the Strengthening the Reporting of Observational Studies in Epidemiology guidelines and Declaration of Helsinki. It was approved by the relevant Ethics Committee (approval date and approval number: October 22, 2024; 2024-0274, study period from data access and extraction permission to completion of paper acceptance: October 22, 2024, to December 31, 2025). Informed consent was obtained from patients using the opt-out method of enrollment via the hospital’s website. Patient data was last accessed for research purposes on November 15, 2024. Subsequently, analysis was carried out using only anonymized numerical data.

Patients and study design

All TEG6s measurements obtained in our operating rooms and intensive care units between January 2018 and July 2024 were extracted. Data from adult patients were used. Although demographic variables such as sex, age, weight, height, and BMI do not fundamentally influence viscoelastic testing results in adults, they were nonetheless extracted to allow for comprehensive dataset presentation. However, details of the surgical procedure were not extracted. Therefore, this study only used data extracted from the database of the viscoelasticity test itself. Pediatric patient data were deleted after the viscoelasticity test result data were extracted. Additionally, data for which the items necessary for analysis, such as measurement failures, were not obtained were deleted. Subsequently, blood collection dates and patient IDs were deleted, and anonymized data were used for the final analysis.

Correlations between CRT-R and the following items were analyzed: CRT-MA, CKH-R, CKH-MA, CFF-MA, and TEG-ACT. Data with CRT-R > 5 times the upper limit of normal were excluded as outliers. After removing missing values and CRT-R values of > 5.5 min, the correlations among the CRT-MA, CKH-R, CKH-MA, and CFF-MA and TEG-ACT, and CRT-R were evaluated statistically. Separate correlation analyses were performed with the CRT-R values as independent variables and other laboratory parameters (CRT-MA, CKH-R, CKH-MA, CFF-MA, and TEG-ACT) as dependent variables. Pearson's correlation coefficients and p-values were calculated for each combination. Taking into account the distribution of the raw data, a linear regression model was used for the relationship between the CRT-R and TEG-ACT, whereas an exponential decay model (y = a exp(-b x) + c, where a represents the initial value, b is the decay rate, and c is the asymptotic value) was used for other relationships. The coefficient of determination (R²) was calculated to assess the model fit. The model accuracy was evaluated using the mean absolute error (MAE) and root mean square error (RMSE). To estimate the uncertainty in the CRT-R cutoff values, we employed a bootstrap resampling method with 1,000 iterations. For each iteration, random samples were drawn with replacement from the original dataset, and the exponential decay model was refitted to calculate the cutoff value. The 25th, 50th (median), and 75th percentiles of the bootstrap distribution were used to estimate the interquartile range, while the 2.5th and 97.5th percentiles were used to estimate the 95% confidence interval of the cutoff values. For each analysis result, a graph that included a scatter plot and a fitted model curve was prepared. Data analysis was performed using Python 3.10.10 (https://www.python.org/).

## Results

During the study period, a total of 2,453 paired measurements of CRT-R with CRT-MA, CKH-R, CKH-MA, CFF-MA, and TEG-ACT were initially identified. Of these, 104 pairs were excluded due to missing values (n = 80) or CRT-R values > 5.5 min (n = 24), leaving 2,349 paired measurements from adult patients undergoing cardiac surgery with cardiopulmonary bypass (CPB) for analysis.

The mean age was 63.1 years (SD: 15.5; range: 10-92), and the cohort consisted of both male and female patients. The mean height was 162.3 cm (SD: 9.9), and the mean body weight was 60.5 kg (SD: 12.8), resulting in a mean body surface area of 1.64 m² (SD: 0.20) and a mean body mass index (BMI) of 22.9 kg/m² (SD: 3.88). Regarding intraoperative parameters, the mean operating room time was 463.4 minutes (SD: 157.6), and the mean anesthesia time was 445.6 minutes (SD: 156.5). The mean surgical time was 356.5 minutes (SD: 147.2), with a mean CPB duration of 174.6 minutes (SD: 80.5) and a mean aortic cross-clamp time of 100.0 minutes (SD: 73.1). The median CRT-R was 0.7 min (IQR: 0.5-0.9; range 0.3-5.5), and the median CRT-MA was 54.2 mm (IQR: 47.7-60.3; range 20.1-75.4). For the subset with available ACT data, the median TEG-ACT was 116.0 s (IQR: 97.3-134.7; range 60.0-199.0).

Correlation analyses

**Figure 1:**
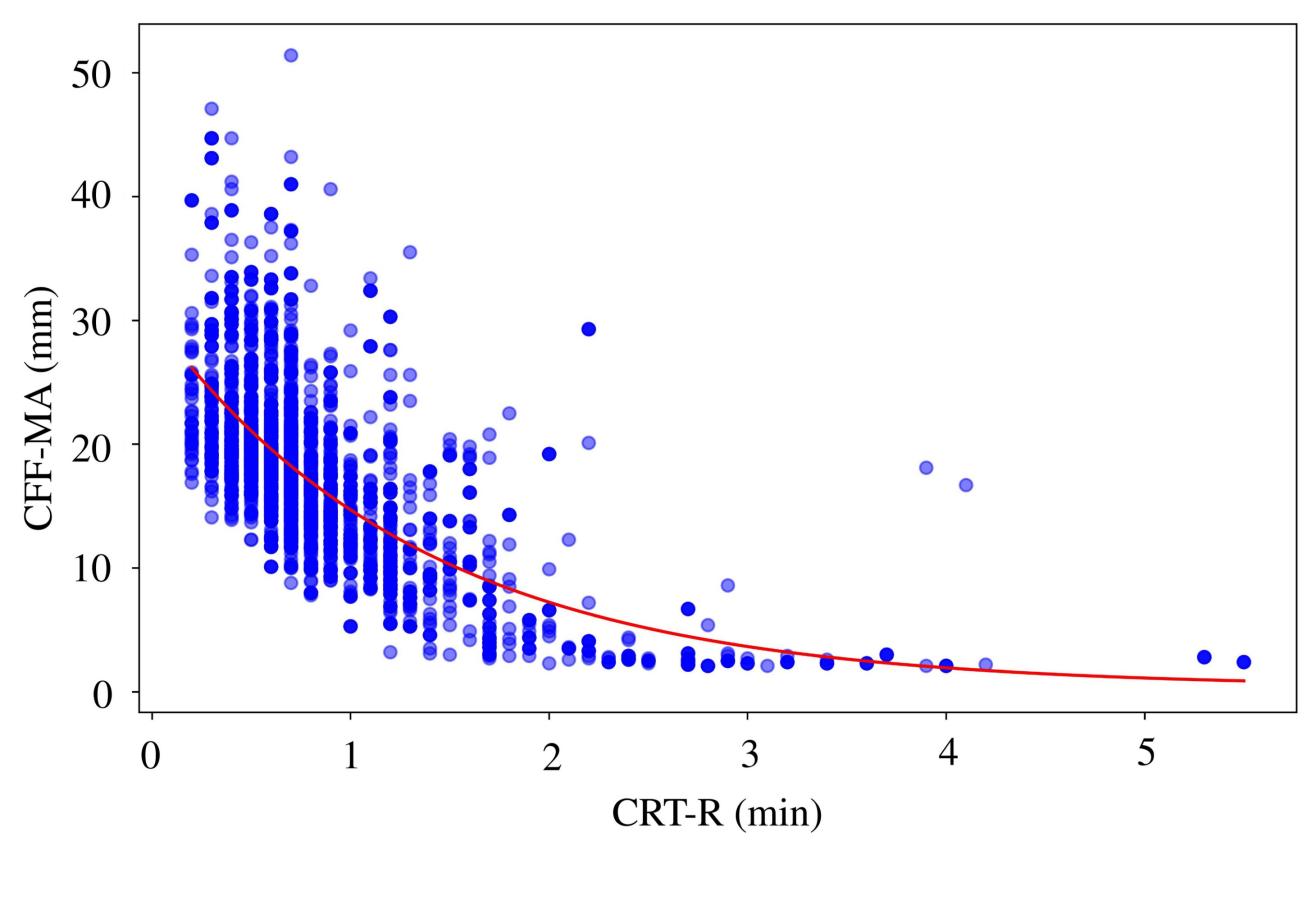
Correlation between the CRT-R and CFF-MA Correlation coefficient = 0.6224 (p < 0.0001), R^2^ = 0.4554, MAE = 3.6584, RMSE = 5.0788, exponential decay model: y = 29.9054 * exp(-0.7345 * x) + 0.3548. TEG, thromboelastography; CRT, citrated rapid TEG; R, reaction time; MA, maximum amplitude; R^2^, coefficient of determination; MAE, mean absolute error; RMSE, root mean square error.

**Figure 2:**
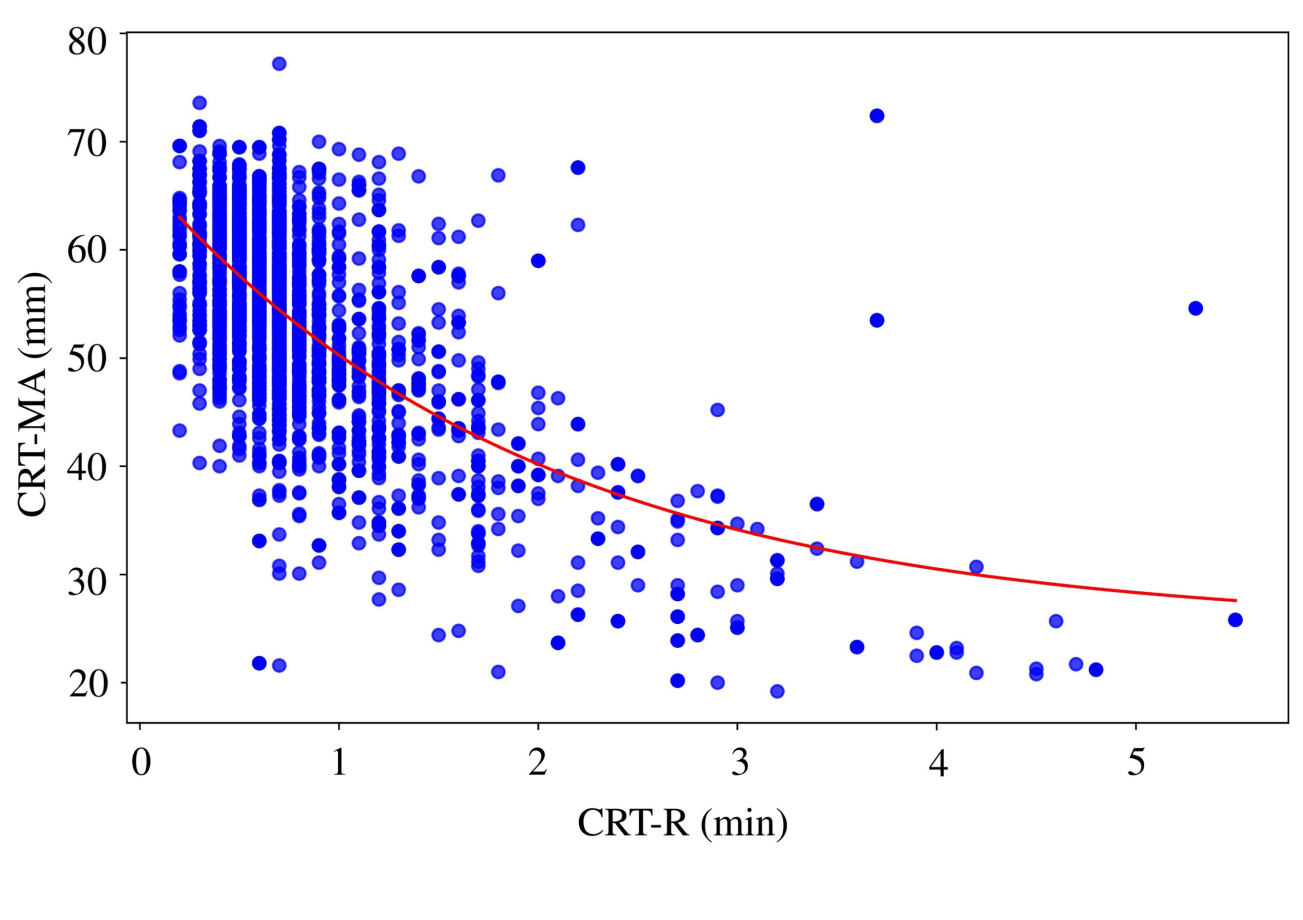
Correlation between CRT-R and CRT-MA Correlation coefficient = 0.6215 (p < 0.0001), R^2^ = 0.4295, MAE = 4.8404, RMSE = 6.5485, exponential decay model: y = 39.4557 * exp(-0.5034 * x) + 27.5760 TEG, thromboelastography; CRT, citrated rapid TEG; R, reaction time; MA, maximum amplitude; R^2^, coefficient of determination; MAE, mean absolute error; RMSE, root mean square error.

**Figure 3:**
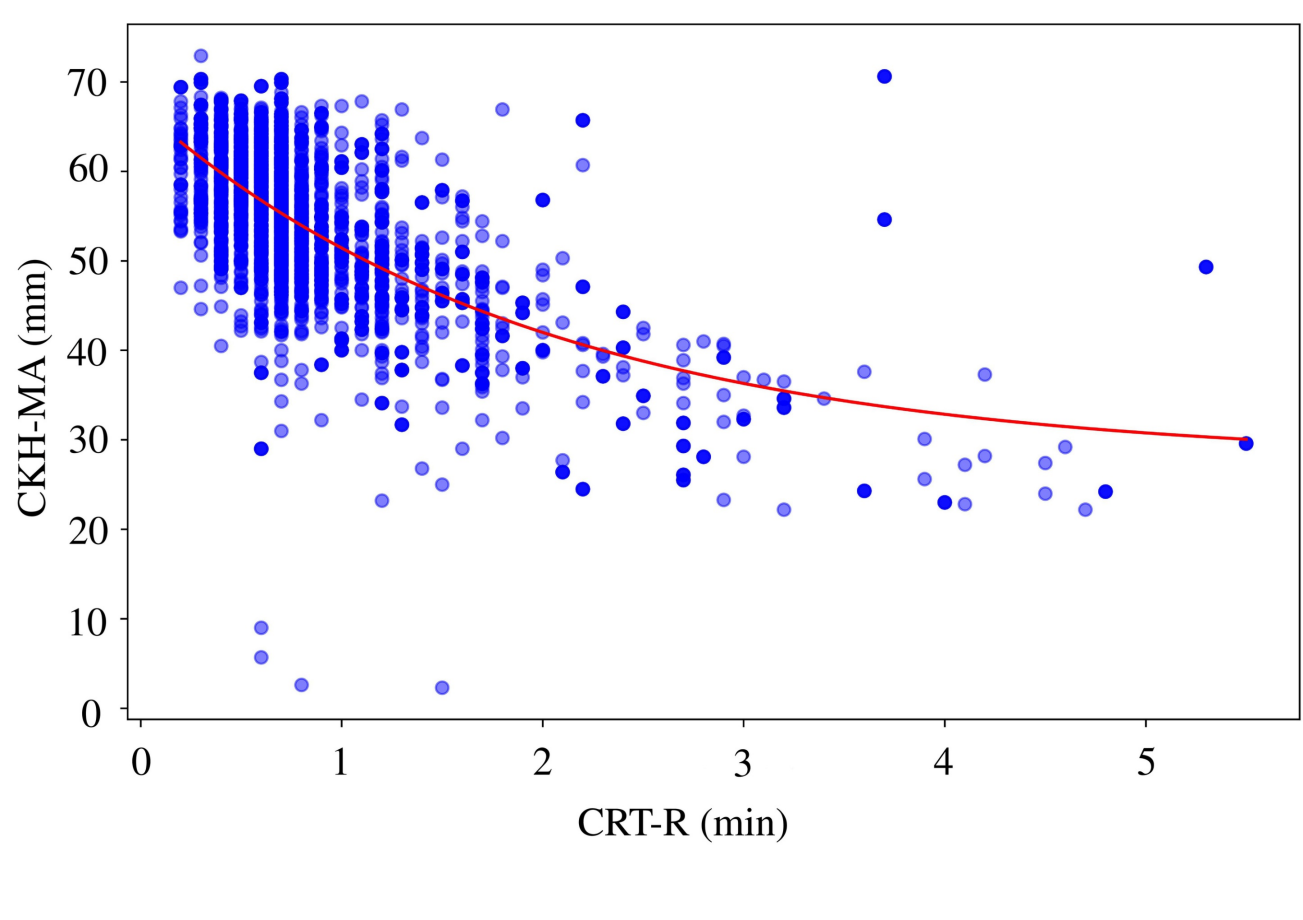
Correlation between CRT-R and CKH-MA Correlation coefficient = 0.6022 (p < 0.0001), R^2^ = 0.4050, MAE = 5.7109, RMSE = 7.4195, exponential decay model: y = 42.0377 * exp(-0.5107 * x) + 25.0521. TEG, thromboelastography; CRT, citrated rapid TEG; R, reaction time; CKH, citrated kaolin with heparinase; MA, maximum amplitude; R^2^, coefficient of determination; MAE, mean absolute error; RMSE, root mean square error.

**Figure 4:**
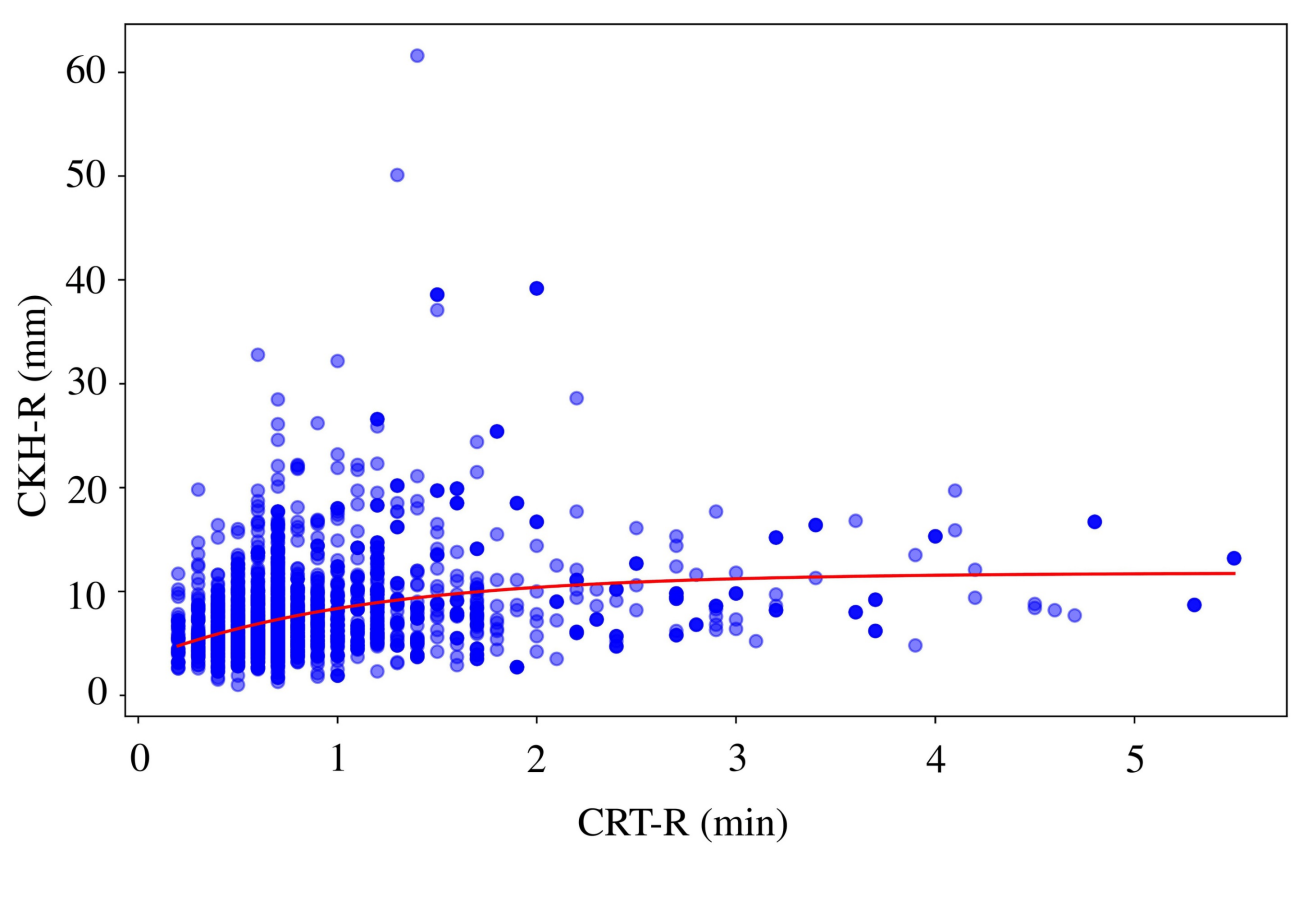
Correlation between CRT-R and CKH-R Correlation coefficient = 0.2897, R^2^ = 0.1080, MAE = 2.5962, RMSE = 3.9846, exponential decay model: y = -8.4209 * exp(-0.8974 * x) + 11.7798. TEG, thromboelastography; CRT, citrated rapid TEG; R, reaction time; CKH, citrated kaolin with heparinase; MA, maximum amplitude; R^2^, coefficient of determination; MAE, mean absolute error; RMSE, root mean square error.

A moderate negative correlation was observed between CRT-R and CFF-MA (r = 0.6224, p < 0.0001), with the highest coefficient of determination (R² = 0.4554) and lowest prediction error (MAE = 3.66, RMSE = 5.08) among all MA parameters (Figure 1). Similar correlations were found between CRT-R and CRT-MA (r = 0.6215, R² = 0.4295; Figure 2) and between CRT-R and CKH-MA (r = 0.6022, R² = 0.4050; Figure 3). In contrast, the correlation between CRT-R and CKH-R was weak (r = 0.2897, R² = 0.1080), indicating poor predictive performance (Figure 4). Figures 1-3 illustrate the inverse relationships between CRT-R and MA values, whereas Figure 4 shows a scattered distribution without a clear trend.

**Figure 5:**
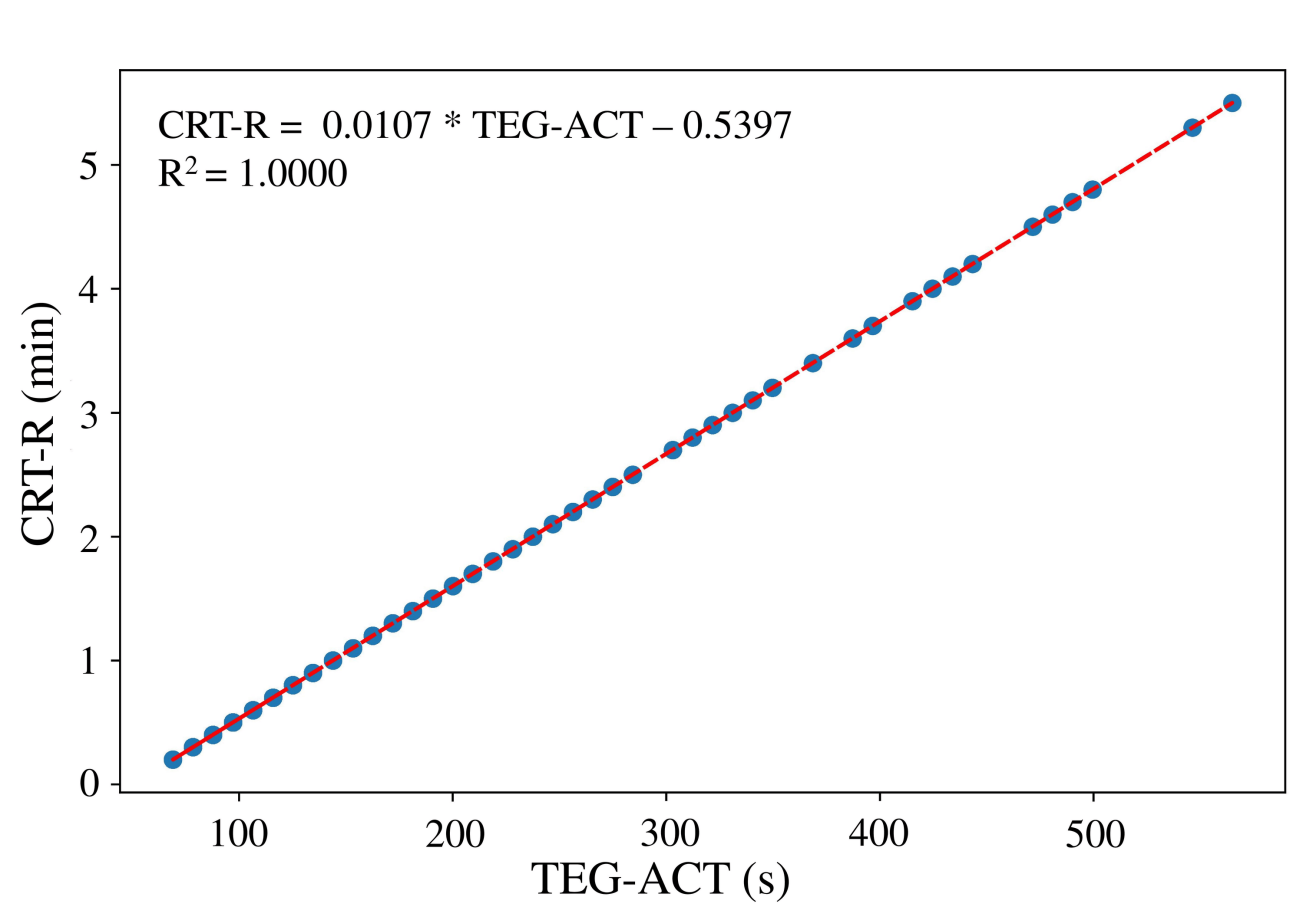
Correlation between CRT-R and TEG-ACT Correlation coefficient = 1.000 (p < 0.0000), R^2 ^= 1.000, MAE = 0.0003, RMSE = 0.0003, exponential decay model: y = 0.0107 * TEG-ACT – 0.5397 TEG, thromboelastography; CRT, citrated rapid TEG; R, reaction time; MA, maximum amplitude; R^2^, coefficient of determination; MAE, mean absolute error; RMSE, root mean square error.

CRT-R and TEG-ACT were linearly and almost perfectly aligned (r = 1.000, p < 0.0001; Figure 5). This alignment reinforces the analytical equivalence of the two metrics, each representing reaction time through distinct measurement systems, most likely via a fixed numerical conversion algorithm embedded within the device.

Cutoff value estimation

**Figure 6:**
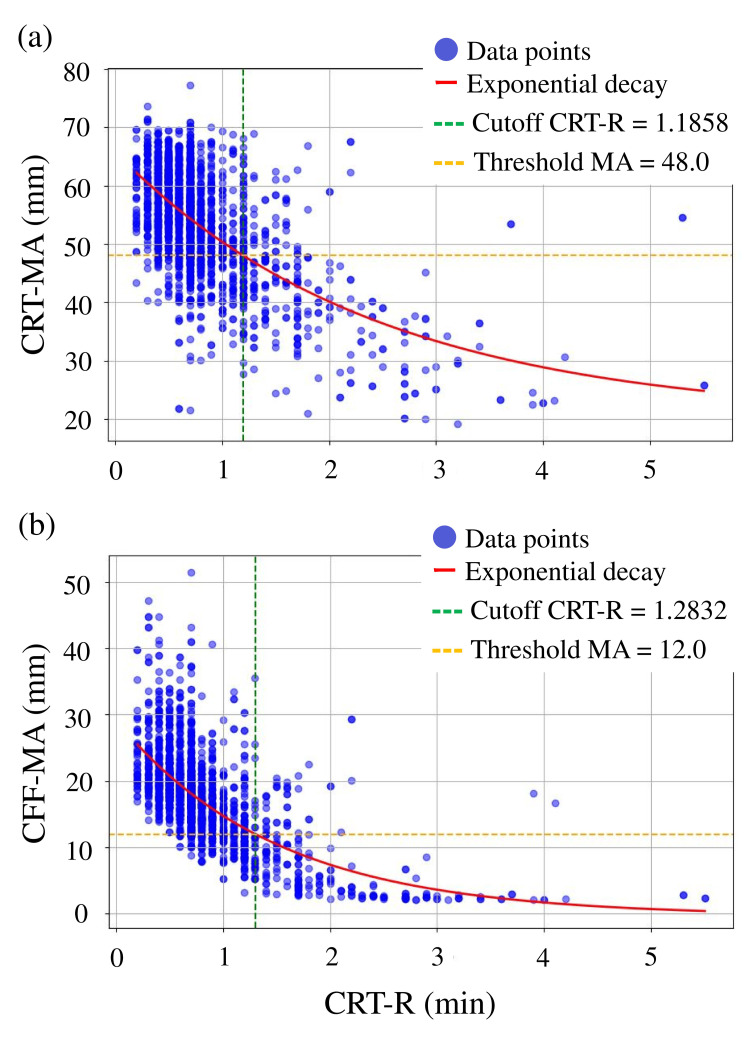
Cutoff value (a) At a CRT-MA = 48, the cutoff value for the CRT-R was 1.20, (b) At a CFF-MA = 12, the cutoff value for the CTR-R was 1.30. TEG, thromboelastography; CRT, citrated rapid TEG; R, reaction time; MA, maximum amplitude

Bootstrap resampling (1,000 iterations) was used to estimate CRT-R thresholds corresponding to clinically relevant MA targets. For CRT-MA = 48 mm [5], the median CRT-R cutoff was 1.1858 min (25-75% CI: 1.1769-1.1955; 95% CI: 1.1597-1.2135). For CFF-MA = 12 mm [6], the median cutoff was 1.2832 min (25-75% CI: 1.2699-1.2961; 95% CI: 1.2487-1.3238) (Figure 6). In Figure 6, the vertical dashed lines denote these thresholds, providing a visual representation of potential early triggers for identifying patients at risk of insufficient platelet- or fibrinogen-related clot strength. When the optimal CRT-R cutoff was applied for predicting CRT-MA <48 mm (1.1858 min), the sensitivity, specificity, positive predictive value, negative predictive value, and accuracy were 49.2%, 93.8%, 72.5%, 84.8%, and 82.7%, respectively. For predicting CFF-MA <12 mm (cutoff 1.2832 min), the corresponding values were 56.9%, 97.0%, 80.3%, 91.4%, and 90.0%, respectively.

## Discussion

This study provides important insights into the role of each test in the blood-clotting process and its interrelationships. Particularly, the strong correlation between the R (min) value of CRT and the MA (mm) value of other tests suggests an inverse relationship between the coagulation R value and its MA, which may be clinically important. The identification of significant correlations between CRT-R and parameters such as CRT-MA, CKH-MA, and CFF-MA suggests that CRT-R, which is available within minutes of initiating the assay, may serve as an effective early predictor of clot quality, particularly the functional contributions of platelets and fibrinogen. The strongest correlation was observed between CRT-R and CFF-MA, emphasizing the early detectability of fibrinogen-related clot strength.

Physiological basis for the CRT-R-MA/CFF-MA relationship

By definition, CRT-R represents the time required for the clot to reach an amplitude of 2 mm, marking the onset of measurable fibrin polymerization. Although CRT-R is classically interpreted as reflecting thrombin generation and overall coagulation factor activity, prior experimental work has demonstrated that its determinants are broader. For example, a seminal in vitro study by Bowbrick et al. [7] showed that while the thromboelastography parameters MA and K are strongly correlated with platelet count and function, the R value exhibits only a weak association with platelet count, indicating that R is more dependent on plasma coagulation factors and fibrin formation kinetics than on platelet contribution.

Furthermore, clinical investigations in specific coagulopathic settings have revealed the influence of fibrinogen on the R time. Huissoud et al. [8] reported that in severe hypofibrinogenemia, the formation of initial fibrin strands is delayed despite adequate thrombin generation, leading to prolonged R times and reduced final clot firmness. This finding supports the concept that fibrinogen availability and quality can indirectly modulate CRT-R values. Conversely, in hypercoagulable states, often characterized by elevated fibrinogen levels and platelet counts, Liu et al. observed shorter clotting times (CTs) and higher MA values, reflecting rapid fibrin polymerization and robust clot strengthening [9].

The stronger correlation between CRT-R and CFF-MA than that between CRT-R and CRT-MA observed in the present study may be explained by the fact that CFF-MA specifically isolates the fibrinogen contribution by pharmacologically inhibiting platelet function. This targeted measurement parallels the fibrinogen-dependent component influencing CRT-R, whereas CRT-MA incorporates both fibrinogen (~20%) and platelet (~80%) contributions [10], the latter of which may be less tightly linked to the timing of clot initiation when platelet function remains preserved.

Comparison with previous literature

Several clinical contexts support these findings. In obstetric hemorrhage, low fibrinogen concentration is associated with both prolonged ROTEM CT and reduced FIBTEM clot amplitude, with strong correlations between fibrinogen and early/maximum amplitudes (r≈0.85) [8]. In trauma, fibrinogen is often the first coagulation factor to fall to critically low levels, and low functional fibrinogen MA has been linked to delayed clot formation, increased transfusion requirements, and poor outcomes [3]. In vitro, fibrinogen concentrate administration has been shown to shorten clot initiation and increase clot strength in parallel [3]. These data align with our observation that CRT-R is inversely correlated with both CFF-MA and CRT-MA in patients after CPB, where hemodilution, consumption, and platelet dysfunction often co-exist.

Our group previously demonstrated that CRT-MA can be predicted from CRT-A10 and that CRT-MA and CFF-A10 are strongly correlated [4]. However, because A10 occurs 10 min after R determination, it remains inherently delayed. This study extends this concept by showing that CRT-R itself, available within minutes of assay initiation, correlates with final MA values, potentially enabling the prediction of platelet- and fibrinogen-related clot strength substantially earlier than existing methods.

Previous trauma studies have shown that a TEG-ACT >128 s predicts massive bleeding within 6 h, whereas <105 s indicates no transfusion requirement [11]. In our dataset, these corresponded to CRT-R values of 0.83 min (49.8 s) and 0.58 min (35.1 s), respectively. Given that CRT-R >0.83 is also associated with low fibrinogen-related clot strength (e.g., CFF-MA <14 [4]), such thresholds could be used to trigger pre-emptive fibrinogen or platelet preparation. Although the correlations between CRT-R and MA values were moderate (r ~ 0.60), their consistent significance and reproducibility suggest a role for CRT-R in predictive algorithms. Bootstrap-derived CRT-R cutoffs in this study may thus inform future transfusion algorithms to improve the timing of hemostatic interventions. However, although the proposed CRT-R cutoffs demonstrated high sensitivity and specificity, a non-negligible proportion of cases with low MA values still existed at these thresholds. This finding highlights the potential risk of overlooking clinically relevant coagulation deficits when using CRT-R in isolation. Therefore, while CRT-R may serve as a rapid screening tool for predicting clot strength, it should be interpreted alongside other viscoelastic parameters and the overall clinical context to avoid underestimating coagulopathy in patients undergoing cardiac surgery.

Clinical implications

During high-risk surgeries, such as cardiac procedures, rapid and accurate identification of coagulation deficits is essential to guide transfusion therapy and reduce bleeding-related complications and unnecessary product administration. Conventional coagulation tests, including prothrombin time and activated partial thromboplastin time, are time-consuming and fail to reflect the integrated functional contributions of platelets and fibrinogen in whole blood [5]. Thromboelastography provides a more comprehensive and dynamic assessment of hemostasis, and the European Guidelines for the Management of Perioperative Bleeding recommend viscoelastic testing as a potential basis for intraoperative coagulation monitoring [1]. However, a recognized limitation is that full maximum amplitude results from thromboelastography or thromboelastometry may take 30-60 min to obtain, which is suboptimal during active bleeding when rapid decisions are critical. Although A10 is widely used in clinical practice owing to its stability and reproducibility, its measurement requires approximately 10-15 min. Nevertheless, CRT-R should be regarded as an early global surrogate marker of clot strength rather than a tool to identify the specific hemostatic component (platelets or fibrinogen) responsible for an abnormal MA value. Contrastingly, CRT-R provides the earliest available signal of clot formation, usually within a few minutes, and may therefore allow more rapid transfusion decision-making in urgent perioperative settings. Thus, while A10 represents a robust and valuable parameter for routine practice, our study focused on CRT-R to explore its potential as an earlier indicator.

By enabling the prediction of MA values within minutes, CRT-R may help bridge this gap. For example, if CRT-R exceeds the threshold predicting low CFF-MA, fibrinogen concentrate preparation could be initiated immediately; if CRT-R predicts low CRT-MA, platelet transfusion could be arranged in advance. Such early activation could shorten turnaround times for targeted transfusion therapy, reduce unnecessary product use, and improve patient outcomes. This study expands upon our prior work by demonstrating that CRT-R, itself available within minutes of assay initiation, correlates with final MA values, potentially enabling the prediction of platelet- and fibrinogen-related clot strength substantially earlier than existing methods. Furthermore, our previously developed transfusion algorithm using TEG6s [12] demonstrated the clinical value of early viscoelastic parameters in guiding targeted hemostatic therapy in the Japanese clinical setting. Incorporating CRT-R-based thresholds into such algorithms could further reduce the time to appropriate product preparation and administration, thereby enhancing the efficiency and precision of perioperative hemostatic management. It is essential to note that the observed correlation between CRT-R and CKH-R was evaluated purely for exploratory purposes. Although a statistical relationship was demonstrated, it has no direct clinical implications. This result should be interpreted as a descriptive finding rather than evidence of predictive clinical utility.

Our study used CRT-R to provide earlier supplementary information in time-sensitive clinical settings and was not intended to replace A10 values. A10 remains a reliable indicator of clot firmness; however, CRT-R may offer additional clinical value by enabling the earlier anticipation of transfusion needs. By acknowledging both its utility and limitations, CRT-R may complement, but not replace, other viscoelastic parameters in guiding timely and appropriate hemostatic therapy. Our findings suggest that CRT-R may serve as a surrogate marker for MA; however, it does not directly reflect platelet function. Therefore, the predictive utility of CRT-R should be regarded as complementary, particularly in the context of fibrinogen supplementation rather than platelet transfusion decision-making.

Limitations

This study has several limitations. First, it was a retrospective analysis. However, we believe that the results of a prospective study would likely yield similar findings. The TEG6s is not a device for directly measuring fibrinogen levels or platelet counts, but rather a whole-blood viscoelastic device for assessing overall clotting capacity [7-9]. Therefore, its results should be interpreted in the context of a comprehensive coagulation assessment rather than as isolated laboratory values. Institutional consensus on transfusion decision-making among surgeons, anesthesiologists, and the transfusion management department is also important, and each institution should consider how the present findings could be adapted to its own transfusion protocols.

Because a transfusion determination method based on the TEG6s algorithm was used in our institution [12], multiple measurements were obtained from the same patient. These measurements were not consecutive; given that coagulation status during and immediately after cardiovascular surgery is prone to variability and that sampling was performed at time points separated by several hours, we assumed that the independence of repeated measurements was preserved. In addition, we observed a relatively wide dispersion of CRT-MA and CFF-MA values corresponding to certain CRT-R ranges. This variability indicates that CRT-R cannot fully capture interpatient differences in clot strength and should therefore be interpreted as an approximate predictor rather than a precise surrogate.

Finally, although patient characteristics, such as procedure type, age, and body weight, were recorded, the transfusion algorithm based on TEG6s used in our clinical practice was applied uniformly, regardless of these variables. Accordingly, patient background factors were not incorporated into the dataset for primary correlation analyses. Preliminary subgroup assessments did not indicate a clinically relevant impact of these variables on the relationship between CRT-R and MA values; nevertheless, validation in larger and more heterogeneous populations is warranted to confirm the generalizability of our findings.

## Conclusions

In this single-center observational study of adult patients who underwent cardiac surgery with CPB, CRT-R demonstrated moderate inverse correlations with CRT-MA, CKH-MA, and CFF-MA, with the strongest association observed for CFF-MA. These findings suggest that CRT-R, which becomes available within minutes of test initiation, may enable the rapid estimation of both fibrinogen- and platelet-related clot strength well before full MA results are reported. Such early predictive capability could facilitate timely preparation and administration of targeted hemostatic therapy, potentially reducing bleeding-related complications and avoiding unnecessary transfusions. While further validation in prospective multicenter settings is warranted, particularly to account for diverse surgical populations and perioperative protocols, the present results support the incorporation of CRT-R thresholds into intraoperative transfusion algorithms as a practical and efficient adjunct to comprehensive viscoelastic assessment.
